# *Francisella tularensis* ssp. *holarctica* in Ringtail Possums, Australia

**DOI:** 10.3201/eid2307.161863

**Published:** 2017-07

**Authors:** John-Sebastian Eden, Karrie Rose, Jimmy Ng, Mang Shi, Qinning Wang, Vitali Sintchenko, Edward C. Holmes

**Affiliations:** University of Sydney, Sydney, New South Wales, Australia (J.-S. Eden, M. Shi, V. Sintchenko, E.C. Holmes);; Westmead Institute for Medical Research, Westmead, New South Wales, Australia (J.-S. Eden, M. Shi);; Australian Registry of Wildlife Health, Mosman, New South Wales, Australia (K. Rose);; James Cook University, Townsville, Queensland, Australia (K. Rose);; Westmead Hospital, Westmead (J. Ng, Q. Wang, V. Sintchenko)

**Keywords:** tularemia, Francisella tularensis, ringtail possum, Pseudocheirus peregrinus, Australia, metagenomics, next-generation sequencing

## Abstract

The occurrence of *Francisella tularensis* outside of endemic areas, such as North America and Eurasia, has been enigmatic. We report the metagenomic discovery and isolation of *F. tularensis* ssp. *holarctica* biovar *japonica* from diseased ringtail possums in Sydney, Australia. This finding confirms the presence of *F. tularensis* in the Southern Hemisphere.

Tularemia is a highly infectious zoonotic disease caused by the bacterium *Francisella tularensis* that affects humans and other animals (*1**,**2*). Globally, tularemia has been identified in a wide range of animal hosts; rabbits and rodents are the most important reservoirs (*3*). Tularemia in humans is typically acquired through direct exposure to infected animals during hunting, although biting insect vectors such as ticks and mosquitos, as well as waterborne and environmental sources, have been reported (*1*). Outbreaks are not uncommon in disease-endemic areas, and a positive correlation exists between the population density of the animal reservoir and the number of human tularemia cases (*4*). 

Ulceroglandular tularemia is the most common form of disease in humans, accounting for ≈75% of reported cases, and is characterized by fever, ulceration at the site of infection, and enlargement of local lymph nodes (*5*). Untreated infection by *F. tularensis* can be life-threatening. Given its multiple routes of transmission, including inhalation of contaminated dust, the bacterium is considered a category A bioterrorism agent in most jurisdictions.

Tularemia is endemic to parts of the Northern Hemisphere, including North America and Eurasia, and most cases in humans are caused by the *F. tularensis* subspecies *tularensis* (type A) and *holarctica* (type B). Notably, regions in the Southern Hemisphere, including Australia, have been largely considered tularemia-free. In 2003, a divergent *Francisella* spp. was isolated from a human foot wound in the Northern Territory, Australia (*6*), and has since been reclassified as *F. hispanensis* (*7*). However, in 2011, a case of ulceroglandular tularemia was reported in an adult bitten by a wild ringtail possum (*Pseudocheirus peregrinus*) in western Tasmania, Australia (*8*). No isolate was obtained in this case, although infection by *F. tularensis* was suggested by both 16S rRNA sequencing and serology (*8*). Two additional cases of suspected human tularemia were reported in Tasmania in 2011, close to the site of the original infection; 1 of these involved exposure to ringtail possums (*9*). Together these cases suggest a possible wider distribution of *F. tularensis* in the Southern Hemisphere and a potential reservoir in ringtail possums in Australia.

## The Study

As part of a wider study of neglected and undiagnosed disease syndromes observed in Australia wildlife, we investigated the possible infectious cause of several deaths in ringtail possums that were most often associated with acute necrotizing enteritis or hepatitis. In total, 8 possums were included in this study, each representative of a distinct mortality event in Sydney, New South Wales, during 2000–2009. Liver and brain tissue were collected at the time during necropsy and stored at −80**°**C.

To identify potential pathogens in these possum samples, we used an unbiased, high-throughput RNA sequencing approach (RNA-Seq; Epicentre, Madison, WI, USA). Total RNA was first extracted from the 8 affected liver tissues and pooled before host ribosomal RNA depletion and library preparation by using Ribo-Zero Gold (Illumina; San Diego, CA, USA) with TruSeq Stranded mRNA Library preparation kit (Illumina). The library was sequenced by using the Illumina HiSeq2500 platform, producing 50,740,088 paired-end sequence reads (100 nt lengths). These data were assembled de novo using Trinity (*10*) and screened for viral, bacterial, and fungal pathogens by comparisons to existing databases by using nucleotide and protein Blast searches (*11*).

No viral or fungal pathogens were apparent in the de novo assembled transcripts. Strikingly, however, *F. tularensis* was abundant, representing ≈85% of the bacterial contigs identified. Other, less abundant bacteria were species from the genera *Serratia*, *Streptococcus*, *Escherichia*, and *Bacillus*. Remapping the RNA-Seq data against the complete genome of the *F. tularensis* subsp. *holarctica* reference strain T01 (GenBank accession no. NZ_CP012092) produced an assembly of 80,516 reads, providing 46.9% genome coverage (891,561/1,899,676 nt) at a mean depth of 4.2× and pairwise identity of 99.8%. This relatively high abundance suggested that *F. tularensis* subsp. *holarctica* RNA was probably present in the pooled possum liver sample.

To confirm the infection, individual liver samples were independently screened for *F. tularensis* at the Emerging Infections and Biohazard Response Unit (EIBRU) at Pathology West, Westmead Hospital, Sydney. This screening involved culturing on enriched cysteine heart agar blood culture medium with antibiotic supplementation (*12*), and assays provided through the US Centers for Disease Control and Prevention’s Laboratory Response Network that included direct fluorescent antigen (DFA) testing and real-time PCR. No serum was available for serologic testing. Of the 8 liver samples present in the RNA-Seq pool, 2 were positive (samples 2 and 7) for *F. tularensis* by both DFA and PCR (all 3 gene targets, FT1, FT2, and FT3, were positive). Both of these *F. tularensis*–positive samples were obtained from ringtail possums found in the Sydney north shore area and were associated with 2 separate mass mortality events in May 2002 (sample 7) and August 2003 (sample 2). An *F. tularensis* isolate was also obtained from sample 7 (denoted FT7) that was identified by morphology, biochemical tests, mass spectrometry, and confirmatory testing by DFA and real-time PCR. Whole-genome sequencing was performed on an Illumina NextSeq500 by using Nextera XT libraries prepared from FT7 genomic DNA in addition to control stocks held by the Emerging Infections and Biohazard Response Unit to eliminate them as possible sources of contamination. Raw sequence data are available from the National Center for Biotechnology Information (BioSample accession no. SAMN06114577). 

**Figure 1:**
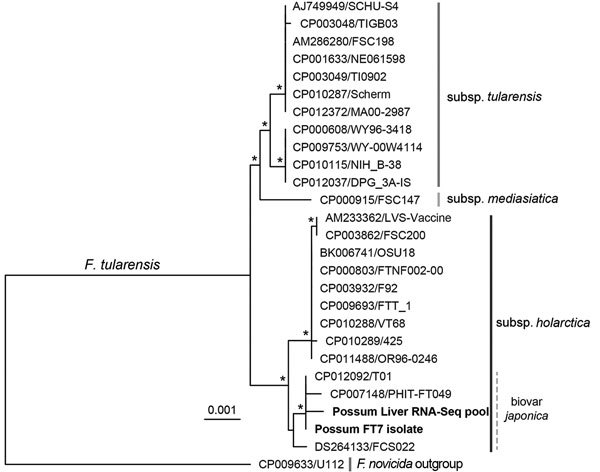
Multilocus phylogenetic analysis of *Francisella tularensis* isolates, including ringtail possum sequences from Australia. The alignment comprised 5 concatenated housekeeping genes (*LepA*, *RecA*, *GyrB*, *AtpD*, and *TrpB*) from the ringtail possum FT7 isolate and RNA-Seq pool (boldface) as well as National Center for Biotechnology Information whole-genome reference sequences (27 sequences, alignment length 7,009 nt). The coverage of each gene from the RNA-Seq data was 71.8%–100%, with mean depths of 1.4×–4.4×. Phylogenetic analysis was performed by using the maximum-likelihood method in PhyML (Hasegawa-Kishino-Yano plus gamma substitution model with 1,000 bootstrap replicates) and rooted by using a *F. novicida* isolate (U112). Isolates are labeled with GenBank accession number and name, and subspecies are indicated. Scale bar indicates number of substitutions per site, and asterisks represent nodes supported by bootstrap values >70%.

**Figure 2:**
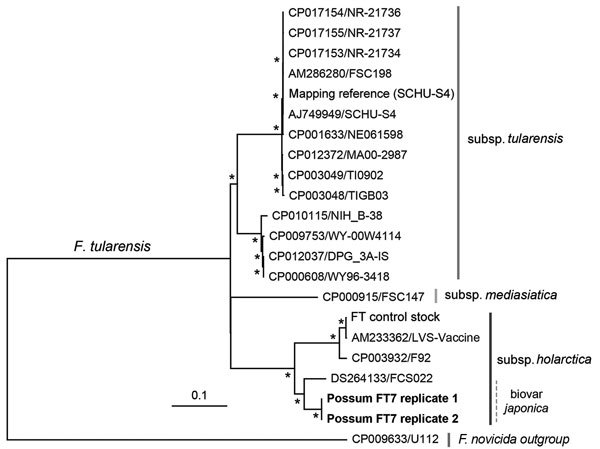
Maximum-likelihood phylogeny of whole-genome single nucleotide polymorphisms of the FT7 *Francisella tularensis* isolate from a ringtail possum in Australia (boldface) with other *Francisella* species, including biovar *japonica*. The single nucleotide polymorphism analysis was performed by mapping reads against a reference genome sequence *F. tularensis* subsp. *tularensis* SCHU-S4 (GenBank accession no. NC_006570.2) in addition to 16 genomes of the *F. tularensis* complex obtained from GenBank. Isolates are labeled with GenBank accession number and name, and subspecies are indicated. Scale bar indicates number of substitutions per site, and asterisks represent nodes supported by bootstrap values >70%.

A multilocus phylogenetic comparison of the FT7 isolate and the original possum liver RNA-Seq data revealed that the sequences clearly clustered together within an Asian subclade of the *holarctica* subspecies that includes biovar *japonica* (Figure 1). The differences between the sequences (3 nt across the multilocus region of 7,009 nt) are probably best explained by sequence quality with poor coverage (only 1×) in the RNA-Seq data at these positions. A subsequent whole-genome single nucleotide polymorphism analysis (Figure 2) and subspecies-specific PCR producing an 839-bp product confirmed FT7 as a member of biovar *japonica* (*13*). Although it is difficult to draw conclusions from such a conserved region, we note that the sequences from the FT7 isolate and the RNA-Seq data both matched, with 100% identity, the 16S rRNA and *recA* gene sequences identified in the human tularemia case in Tasmania (*8*). Testing of additional archived tissues from 8 similarly affected ringtail possums and 3 rabbits did not identify further cases positive for *F. tularensis*. Hence, there are probably multiple etiologies for the acute necrotizing enteritis or hepatitis observed.

## Conclusions

The clinical, gross, and histologic findings, in addition to the genetics and microbiology, confirm the diagnosis of *F. tularensis* infection in >2 ringtail possums associated with 2 mortality events in Sydney. Native ringtail possums in Australia might therefore be a natural reservoir or end-stage host of tularemia and serve as useful sentinels of disease activity that could pose a threat to human health through occasional exposure. A better understanding of the ecology of *F. tularensis* subsp. *holarctica* in Australia is necessary to contribute to public health and to wildlife welfare and conservation.
